# Targeting Intra-Pulmonary P53-Dependent Long Non-Coding RNA Expression as a Therapeutic Intervention for Systemic Lupus Erythematosus-Associated Diffuse Alveolar Hemorrhage

**DOI:** 10.3390/ijms22136948

**Published:** 2021-06-28

**Authors:** Yi-Cheng Chen, Yu-Chi Chou, Yu-Tung Hsieh, Pin-Yu Kuo, Mei-Lin Yang, Hao-Earn Chong, Chao-Liang Wu, Ai-Li Shiau, Chrong-Reen Wang

**Affiliations:** 1 Department of Internal Medicine, Medical College and Hospital, National Cheng Kung University, Tainan 70403, Taiwan; s58971081@gs.ncku.edu.tw (Y.-C.C.); haoen1986@gmail.com (H.-E.C.); 2 Department of Medical Research, Ditmanson Medical Foundation, Chiayi Christian Hospital, Chiayi 600566, Taiwan; 3 Department of Biochemistry and Molecular Biology, National Cheng Kung University Medical College, Tainan 70403, Taiwan; wumolbio@mail.ncku.edu.tw; 4 Biomedical Translation Research Center, Academia Sinica, Taipei 11529, Taiwan; chou0315@gate.sinica.edu.tw; 5 Department of Microbiology and Immunology, Medical College, National Cheng Kung University, Tainan 70403, Taiwan; c216439@gmail.com (Y.-T.H.); fishwukuo012057@gmail.com (P.-Y.K.); ornas331@yahoo.com.tw (M.-L.Y.); alshiau@mail.ncku.edu.tw (A.-L.S.)

**Keywords:** systemic lupus erythematosus-associated diffuse alveolar hemorrhage, p53-dependent apoptosis, long non-coding RNA, short hairpin RNA, intra-pulmonary delivery

## Abstract

Diffuse alveolar hemorrhage (DAH) in systemic lupus erythematosus (SLE) is associated with significant mortality, requiring a thorough understanding of its complex mechanisms to develop novel therapeutics for disease control. Activated p53-dependent apoptosis with dysregulated long non-coding RNA (lncRNA) expression is involved in the SLE pathogenesis and correlated with clinical activity. We examined the expression of apoptosis-related p53-dependent lncRNA, including H19, HOTAIR and lincRNA-p21 in SLE-associated DAH patients. Increased lincRNA-p21 levels were detected in circulating mononuclear cells, mainly in CD4+ and CD14+ cells. Higher expression of p53, lincRNA-p21 and cell apoptosis was identified in lung tissues. Lentivirus-based short hairpin RNA (shRNA)-transduced stable transfectants were created for examining the targeting efficacy in lncRNA. Under pristane stimulation, alveolar epithelial cells had increased p53, lincRNA-p21 and downstream Bax levels with elevated apoptotic ratios. After pristane injection, C57/BL6 mice developed DAH with increased pulmonary expression of p53, lincRNA-p21 and cell apoptosis. Intra-pulmonary delivery of shRNA targeting lincRNA-p21 reduced hemorrhage frequencies and improved anemia status through decreasing Bax expression and cell apoptosis. Our findings demonstrate increased p53-dependent lncRNA expression with accelerated cell apoptosis in the lungs of SLE-associated DAH patients, and show the therapeutic potential of targeting intra-pulmonary lncRNA expression in a pristane-induced model of DAH.

## 1. Introduction

Diffuse alveolar hemorrhage (DAH) in systemic lupus erythematosus (SLE) is a critical condition characterized by the intra-alveolar accumulation of red blood cells (RBCs), resulting in acute respiratory failure [1]. This life-threatening manifestation was firstly described by Sir William Osler in his long-term follow up of SLE patients more than one century ago [2]. Pulmonary capillaritis is the most common histopathological findings of DAH with neutrophilic infiltration of the capillaries, mediated by immune complexes (ICs) with abundant granular electron-dense materials and immunoglobulins (Igs)/C3 deposition along the alveolar and vascular walls [1,3]. Despite intensive respiratory support with mechanical ventilation or plus extra-corporeal membrane oxygenation, based on 10 recent large-scale series including our 17-case retrospective study (Table 1), SLE-associated DAH has a significant fatality with an average mortality rate around 40% [4,5,6,7,8,9,10,11,12,13]. These clinical observations have implicated a more effective control of underlying disease activity to avoid the DAH-related fatality.

The pathogenesis of SLE is characterized by an imbalance between production of apoptotic cells and clearance of apoptosis-derived remnants with the ICs formation containing modified nuclear autoantigens, leading to diffuse visceral inflammation [14]. For SLE-related lung involvement, the DAH manifestation as the consequence of pulmonary capillaritis, is a leading cause of mortality [1]. Novel therapeutic approach to suppress the accelerated apoptosis of pulmonary parenchymal cells including alveolar wall cells and immune effectors like phagocytic macrophages/monocytes and T cells in SLE-associated DAH [1,3,15,16], could reduce the formation of ICs through inhibiting the production of nuclear autoantigens, and restore the disposal of apoptotic materials by abolishing the apoptosis of phagocytes [14]. In a mouse model of DAH, the therapeutic potential by reducing pulmonary cell apoptosis has been demonstrated recently [17]. Besides apoptosis, other cellular death processes comprising autophagy, necroptosis, necrosis, NETosis and pyroptosis are involved in the generation of nuclear autoantigens for the development of SLE [14]. Furthermore, a pathogenic role of NETosis has been identified in the DAH manifestation of SLE [1,3,18].

Long noncoding RNA (lncRNA), transcribed RNA molecules larger than 200 nucleotides without open reading frames and protein-coding capacity, can modulate a variety of cellular activities through the interaction with other intra-nuclear molecules [19]. Increased expression of lncRNA has been demonstrated to be correlated with the SLE clinical activity, and these molecules participate in targeting different signaling transduction pathways like p53-dependent apoptosis [20,21]. In SLE patients, higher p53 levels correlated with their activity were detected in circulating mononuclear cells (MNCs) [22], and a positive correlation existed between nuclear p53 staining of apoptotic cells and glomerular activity indices in renal biopsied specimens [23], indicating an involvement of activated p53-dependent apoptosis in the disease flare-up. Importantly, different cellular effects of p53 can be mediated through the induction of lncRNA, serving as the p53 effector [24]. In addition to apoptosis, lncRNA has been shown to be involved in other cell death processes like autophagy [25], raising a possibility that these molecules could participate in clinical activity and bring about various disease manifestations by simultaneously regulating different death-related programs in SLE patients.

In this study, we examined the expression of apoptosis-related p53-dependent lncRNA, including H19, HOTAIR and lincRNA-p21 [26,27,28], in circulating MNCs and urine cells from SLE-associated DAH patients and sex/age-matched health control (HC) subjects. The expression of p53, lncRNA and cell apoptosis were investigated in lung tissues from DAH patients and pneumothorax (PTX) controls. A pristane-induced DAH model of murine lupus was investigated for the expression of p53, lncRNA and cell apoptosis in the lungs and spleen, as well as CD4+ T cells. Alveolar epithelial cells were examined for lncRNA expression by pristane stimulation to induce cell apoptosis. Lentivirus (LV)-based short hairpin RNA (shRNA)-transduced stable transfectants were created to screen the lncRNA targeting efficacy for in-vivo experiments. Finally, pristane-injected DAH mice received intra-pulmonary delivery of shRNA targeting lncRNA to evaluate the therapeutic efficacy in hemorrhage frequencies and anemia status.

## 2. Results

### 2.1. LncRNA Expression in Circulating MNCs and Urine Cells from SLE-Associated DAH Patients

Firstly, circulating MNCs and urine cells from SLE patients and HC subjects were examined for the expression of p53-dependent lncRNA, lincRNA-p21, HOTAIR and H19.

**Figure 1:**
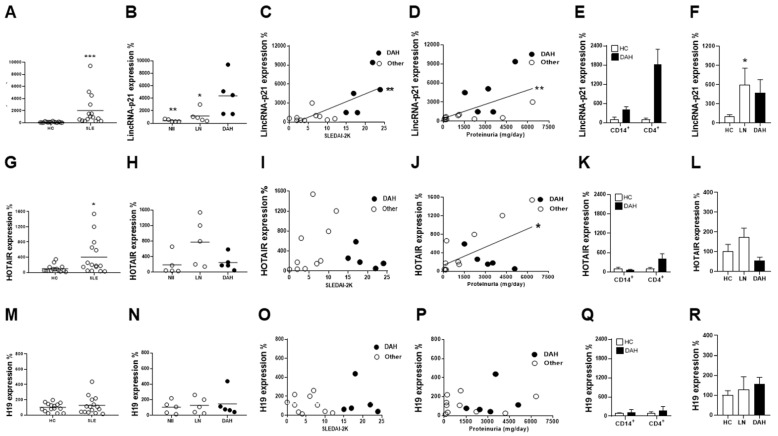
Apoptosis-related p53-dependent lncRNA levels in circulating MNCs, their subpopulations and urine cells from SLE patients with DAH, LN or Nil groups and age/sex-matched HC subjects. (**A**) LincRNA-p21 levels in MNCs from HC subjects and SLE patients. (**B**) LincRNA-p21 levels in MNCs from SLE patients with DAH, LN or Nil. (**C**,**D**) Positive correlations between lincRNA-p21 levels and SLEDAI-2K scores or daily proteinuria amounts. (**E**) LincRNA levels in CD4+ and CD14+ subpopulations of MNCs. (**F**) LincRNA-p21 levels in urine cells from LN and DAH patients and HC subjects. (**G**) HOTAIR levels in MNCs from HC subjects and SLE patients. (**H**) HOTAIR levels in MNCs from SLE patients with DAH, LN or Nil. (**I**,**J**) No correlation between HOTAIR expression and SLEDAI-2K scores, but a positive correlation between its levels and daily proteinuria amounts. (**K**) HOTAIR levels in CD4+ and CD14+ subpopulations of MNCs. (**L**) HOTAIR levels in urine cells from LN and DAH patients and HC subjects. (**M**) H19 levels in MNCs from HC subjects and SLE patients. (**N**) H19 levels in MNCs from SLE patients with DAH, LN or Nil. (**O**,**P**) No correlation between H19 levels and SLEDAI-2K scores or daily proteinuria amounts. (**Q**) H19 levels in CD4+ and CD14+ subpopulations of MNCs. (**R**) H19 levels in urine cells from LN and DAH patients and HC subjects. Relative abundance of a measured gene expression was normalized by GAPDH gene from each sample. The average levels of MNCs or urine cells from HC subjects were determined as 100%. Horizontal lines in (**A**,**B**,**G**,**H**,**M**,**N**) are mean values from HCs and patients. *n* = 15 for HC subjects, *n* = 15 for SLE patients, *n* = 5 for DAH patients, *n* = 5 for LN patients and *n* = 5 for Nil patients. For lncRNA levels in CD4+ and CD14+ subpopulations of MNCs, *n* = 3 for HC subjects and *n* = 2 for DAH patients. For lncRNA levels in urine cells, *n* = 10 for HC subjects, *n* = 5 for LN patients and *n* = 3 for DAH patients. * *p* < 0.05, ** *p* < 0.01, *** *p* < 0.001.

Significantly higher lincRNA-p21 levels in MNCs were found in SLE patients than in HC subjects (Figure 1A, *p* < 0.001). For lincRNA-p21 expression in different patient groups (Figure 1B), DAH patients had higher levels than those with lupus nephritis (LN, *p* = 0.032) or those without DAH or LN (Nil, *p* = 0.008). In addition, DAH patients had higher lincRNA-p21 levels than healthy controls (*p* < 0.001) or those without DAH (*p* = 0.003). A significant positive correlation was found between lincRNA-p21 levels and SLEDAI-2K scores (Figure 1C, r = 0.706, *p* = 0.004) or daily proteinuria amounts (Figure 1D, r = 0.686, *p* = 0.006).

Despite significantly higher levels in MNC HOTAIR expression were found in SLE patients than in HC subjects (Figure 1G, *p* = 0.033), there were no differences among different patient groups (Figure 1H). Significantly higher levels were found in LN patients than in healthy control (*p* = 0.003). Although no correlation existed between HOTAIR expression and activity scores (Figure 1I), a significant positive correlation was found between its levels and proteinuria amounts (Figure 1J, r = 0.565, *p* = 0.028).

No differences were found in MNC H19 expression between SLE patients and HC subjects (Figure 1M) or among different patient groups (Figure 1N). There were no correlations between its levels and activity scores (Figure 1O) or proteinuria amounts (Figure 1P).

Since the accelerated apoptotic pulmonary parenchymal cells in SLE-associated DAH include immune effectors like T cells and monocytes [1,3,15,16], we further examined CD4+ and CD14+ MNC subpopulations for the expression of lincRNA-p21, HOTAIR and H19 from SLE-associated DAH patients and HC subjects. Patients had higher lincRNA-p21 levels in both CD4+ and CD14+ cells (Figure 1E), and higher HOTAIR levels in CD4+ cells (Figure 1K). There were no differences in H19 levels in CD4+ or CD14+ cells between patients and controls (Figure 1Q). In addition, no higher lncRNA expression was found in the CD4/CD14 double-negative subpopulation from DAH patients (data not shown).

Moreover, lncRNA levels were further measured in urine cells from different patient groups and HC subjects. Higher lincRNA-p21 levels were found in LN patients than HC subjects (Figure 1F, *p* = 0.017), while there were no differences in HOTAIR or H19 levels between LN patients and healthy controls (Figure 1L,R). There were no differences in lincRNA-p21, HOTAIR and H19 levels between DAH patients and healthy controls or LN patients (Figure 1F,L,R).

In sum, these results indicated increased lincRNA-p21 expression in circulating MNCs from SLE-associated DAH patients, predominantly in CD4+ and CD14+ cell subpopulations.

### 2.2. Increased Pulmonary p53 and LincRNA-p21 Expression and Cell Apoptosis in SLE-Associated DAH Patients

**Figure 2:**
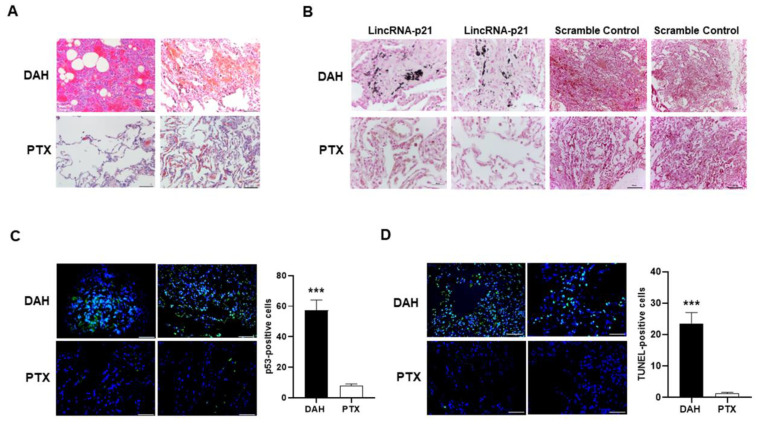
The expression of p53, lincRNA-p21 and apoptotic cells in lung tissues from SLE-associated DAH patients and PTX controls. (**A**) Representative H&E staining of lung tissues (×200) from SLE-associated DAH patients and PTX controls. Bars shown on photomicrographs corresponding to 40 µm. (**B**) Representative ISH expression of lincRNA-p21 analyzed by antisense probe A in lung tissues (dark blue, ×400) from SLE-associated DAH patients and PTX controls as well as negative controls analyzed by scramble probe (×100). Bars shown on ×400 and ×100 photomicrographs corresponding to 20 µm and 100 µm, respectively. (**C**) Representative p53 nuclear expression analyzed by IF stain (left panel, green, ×400) from SLE-associated DAH patients and PTX controls. Number of p53-positive cells (right panel), as determined by averaging the number from 3 fields (×400) of the highest density of positively stained cells in each section. Cell nuclei counterstained with DAPI (blue). Bars shown on photomicrographs corresponding to 20 µm. (**D**) Representative TUNNEL stain with apoptotic cells in lung sections (left panel, green, ×400) from SLE-associated DAH patients and PTX controls. Cell nuclei counterstained with DAPI (blue). Bars shown on photomicrographs corresponding to 20 µm. Number of TUNEL-positive cells (right panel), as determined by averaging the number from 3 fields (×400) of the highest density of positively stained cells in each section. *** *p* < 0.001. *n* = 2 for DAH patients and *n* = 2 for PTX controls.

Figure 2A shows the hematoxylin and eosin (H&E) staining of lung tissues from SLE-associated DAH patients and PTX controls.

Since increased lincRNA-p21 levels were identified in circulating MNCs from SLE-associated DAH patients, we further analyzed its expression in lung tissues by using in situ hybridization (ISH) with two antisense probes, 5′-TGACTTATGATGGTTCAGCTT-3′ (probe A) and 5′-AGGTCTGAAT GTAAGTTGTCT-3′ (probe B). In Figure 2B, distinct lincRNA-p21 expression (dark blue staining) was identified in SLE-associated DAH patients but not in PTX controls by probe A or in negative controls by scramble probe. LincRNA-p21 levels were not increased in circulating MNCs from PTX patients, and there was similar expression with less staining intensity in DAH lung tissues analyzed by probe B (data not shown).

Immunofluorescence (IF) staining was used to detect nuclear p53 expression (green staining) in lung tissues. There were significantly higher numbers of p53-positive cells in SLE-associated DAH patients than in PTX controls (Figure 2C, *p* < 0.001). We further used terminal deoxynucleotidyl transferase dUTP nick end labeling (TUNEL) staining to detect apoptotic cells (green staining). In Figure 2D, there were significantly higher numbers of TNUEL-positive cells in SLE-associated DAH patients than in PTX controls (*p* < 0.001).

### 2.3. LincRNA-p21 Expression in Pristane-Induced Model of DAH and Hydrophilic Pristane-Stimulated Alveolar Epithelial Cells

**Figure 3:**
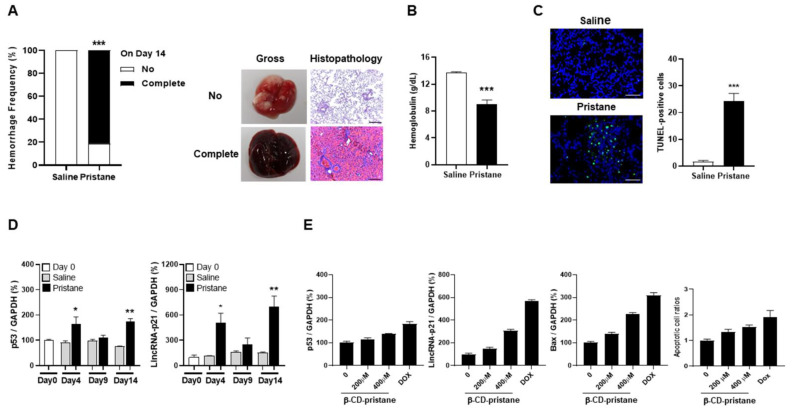
LincRNA-p21 expression in pristane-induced model of DAH and hydrophilic pristane-stimulated alveolar epithelial cells. (**A**) Hemorrhage frequencies in PBS- and pristane-injected C57BL/6 mice on day 14 (left panel). Representative gross and histopathology findings in the lungs with no or complete hemorrhage (right panel). (**B**) Hb levels of PBS- and pristane-injected C57BL/6 mice on day 14. (**C**) Representative TUNNEL staining for apoptotic cells in lung tissues from PBS- and pristane-injected mice (left panel, ×400). Bars shown on photomicrographs corresponding to 20 µm. Numbers of TUNEL-positive cells (right panel), as determined by averaging the number from 3 fields (×400) of the highest density of positively stained cells in each section. (**D**) Serial p53 (left panel) and lincRNA-p21 (right panel) pulmonary expression levels on day 0, 4, 9 and 14 from PBS- and pristane-injected mice. (**E**) p53, lincRNA-p21 and Bax expression as well as apoptotic cell ratios in alveolar epithelial cells stimulated with different concentrations of hydrophilic pristane for 24 h. Relative abundance of a measured gene expression was normalized by GAPDH gene from each sample. The average levels of mouse lung tissues on day 0 and expression levels of alveolar epithelial cells without stimulation were determined as 100%. Values are mean ± SEM. Sixteen mice per group in (**A**,**B**), and 5 mice per group in (**C**,**D**). All of the in-vivo results in (**A**–**D**) and in-vitro results in (**E**) in Figure 3 were representative of two and three independent experiments, respectively, with similar findings. * *p* < 0.05, ** *p* < 0.01, *** *p* < 0.001.

We induced a DAH model in mice by intraperitoneal injection of pristane, and observed the presence of pulmonary hemorrhage by both gross examinations and histopathological findings on day 14 (Figure 3A). Thirteen out of 16 mice developed complete hemorrhage after receiving pristane injection, whereas all of the phosphate-buffered saline (PBS)-injected mice had no hemorrhage (81.3 versus 0%, *p* < 0.001). Furthermore, there were significantly lower hemoglobulin (Hb) levels in pristane-injected mice than PBS-treated controls (Figure 3B, 9.0 ± 0.7 versus 13.7 ± 0.2 g/dL, *p* < 0.001). In addition, proteinuria was not found in mice on day 14. Although RNP antibody levels were not identified on day 14 [29], its levels were detected in 4 out of 16 (25%) pristane-injected mice in the later period, but not in PBS-injected controls.

Significantly increased TUNEL-positive apoptotic cells were found in lung tissues from pristane-injected mice as compared with PBS-treated controls (Figure 3C, *p* < 0.001). p53 and lincRNA-p21 levels were measured in lung tissues on day 0, 4, 9 and 14 after pristane injection. There were increased levels in pristane-injected mice from day 4 onward in comparison with PBS-treated controls with a decrease on day 9 (Figure 3D, p53, day 4, *p* = 0.040, day 14, *p* = 0.001; lincRNA-p21, day 4, *p* = 0.020, day 14, *p* = 0.002).

Alveolar epithelial cells were stimulated with different concentrations of β-cyclodextrin (β-CyD)-conjugated pristane for 24 h, with doxorubicin (Dox) as a positive control to induce p53-dependent cell apoptosis triggered by DNA damage. As shown in Figure 3E, there was a dose-dependent increase in the expression of p53, lincRNA-p21 and downstream Bax. Furthermore, apoptotic cell ratios were dose-dependently increased under the stimulation of hydrophilic pristane.

Collectively, these in-vivo and in-vitro findings suggested that pristane can induce apoptosis in pulmonary cells through increasing the expression of p53-dependent lncRNA.

### 2.4. Induction of CD4+ T Cell Apoptosis with Increased LincRNA-p21 Expression in Pristane-Injected Mice

**Figure 4:**
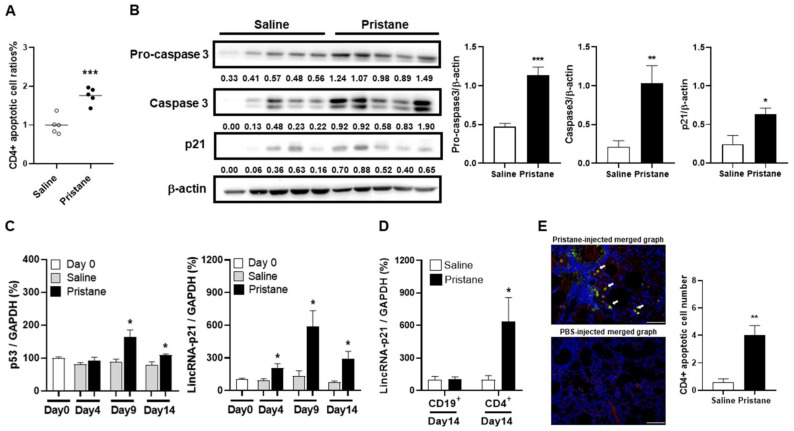
Induction of CD4+ cell apoptosis and apoptosis-related molecules with increased lincRNA-p21 expression in pristane-injected mice. (**A**) CD4+ apoptotic cell ratios on day 14 from PBS- and pristane-injected mice. (**B**) Representative immunoblot assay with quantitation of signal intensity for procaspase 3, caspase 3 and p21 expression in CD4+ splenocytes from PBS- and pristane-injected mice. (**C**) Serial p53 (left panel) and lincRNA-p21 (right panel) splenic expression levels on day 0, 4, 9 and 14 from PBS- and pristane-injected mice. (**D**) LincRNA-p21 expression levels in splenic CD4+ and CD19+ cells on day 14 from PBS- and pristane-injected mice. (**E**) Representative IF staining of TUNEL (green) and CD4 (red) in lung tissues on day 14 from PBS- and pristane-injected mice (left panel, ×400). Cell nuclei counterstained with DAPI (blue). Arrows indicating CD4 and TUNEL double-positive cells in the merged photograph. Bars shown on photomicrographs corresponding to 20 µm. CD4+ apoptotic cell numbers in lung tissues on day 14 from PBS- and pristane-injected mice, as determined by averaging the number from 3 fields (×400) of the highest density of positively stained cells in each section (right panel). Relative abundance of a measured gene expression was normalized by GAPDH gene from each sample. The average levels of mouse splenocytes on day 0 and purified cell subpopulations of PBS-injected mice on day 14 were determined as 100%. Five mice per group in (**A**–**E**) experiments. Values are mean ± SEM. All of the in-vivo and ex-vivo results in Figure 4 were representative of two independent experiments with similar findings. * *p* < 0.05, ** *p* < 0.01, *** *p* < 0.001.

There were significantly higher apoptotic cell ratios in CD4+ cells from pristane-injected mice than from PBS-treated controls on day 14 (Figure 4A, *p* < 0.001). CD4+ cells were isolated from the spleen, and examined for the expression of apoptosis-related molecules, procaspase 3, caspase 3 and p21 by immunoblot assay. There were higher levels in pristane-injected mice than in PBS-treated controls (Figure 4B, procaspase 3, *p* < 0.001, caspase 3, *p* = 0.009, p21, *p* = 0.024). p53 and lincRNA-p21 expression was measured in the spleen on day 0, 4, 9 and 14 after pristane injection. There were increased levels in pristane-injected mice from day 4 onward, as compared with PBS-treated controls with a decrease on day 14 (Figure 4C, p53, day 9, *p* = 0.032, day 14, *p* = 0.016; lincRNA-p21, day 4, *p* = 0.041, day 9, *p* = 0.019, day 14, *p* = 0.018). Moreover, CD4+ cells from pristane-injected spleen on day 14 had significantly higher lincRNA-p21 levels than PBS-treated controls (Figure 4D, *p* = 0.030), while there were no differences in CD19+ cells.

We further analyzed apoptotic CD4+ cells in lung tissues from pristane-injected mice by IF staining. There were significantly higher numbers of CD4 and TUNEL double-positive cells in pristane-injected mice than in PBS-treated controls (Figure 4E, *p* = 0.002).

Taken together, these data demonstrated that increased lincRNA-p21 expression could induce apoptosis of CD4+ cells in pristane-injected mice.

### 2.5. LV-Based shRNA Targeting LincRNA-p21 Efficacy Screening

**Figure 5:**
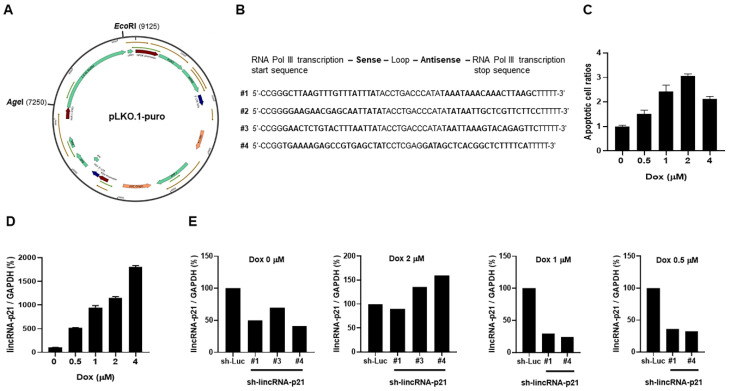
pLKO.1-puro and lincRNA-p21 targeting efficacy in sh-lincRNA-21-transduced stable transfectants. (**A**) Map of pLKO.1-puro with a 1.9 kb stuffer removed by AgeI and EcoRI cutting, a total of 9.3 kb in length. (**B**) Four designed shRNA sequences targeting mouse lincRNA-p21. (**C**,**D**) Apoptotic cell ratios and lincRNA-p21 expression levels in transfectants stimulated with various concentrations of Dox for 24 h. (**E**) Representative lincRNA-p21 targeting efficacy in three sh-lincRNA-p21-transfected transfectants with sh-luciferase-transfected transfectant as the control under different concentrations of Dox stimulation. Relative abundance of a measured gene expression was normalized by GAPDH gene from each sample. The expression levels of alveolar epithelial cells without stimulation or control transfectants were determined as 100%. Values are mean ± SEM. All of the in-vitro results in Figure 5 were representative of at least three independent experiments with similar findings.

Figure 5A shows the pLKO.1-puro vector, 9.3 kb in length. After removing a 1.9 kb stuffer, four designed shRNA sequences targeting mouse lincRNA-p21 (Figure 5B) were cloned into this vector to create LV-sh-lincRNA-p21. Alveolar epithelial cells were stimulated with Dox, resulting in a dose-dependent increase in apoptotic cell ratios (Figure 5C) and lincRNA-p21 levels (Figure 5D). These cells were further transfected with four created recombinant LV vectors to produce three stable transfectants by puromycin selection process (#1, #3, #4 survivable, #2 not survivable), and were examined for the knockdown efficacy in lincRNA-p21 expression without and under the stimulation with 0.5, 1 and 2 µM Dox. The results in Figure 5E were a representative of at least three independent experiments with similar findings. There was lower efficacy in #3, and #3 or #4 failed to reduce lincRNA-p21 expression in the presence of 2 µM Dox to induce the maximal apoptotic cell ratios.

From the screening results, we used sh-lincRNA-p21 LV vector with #1 targeting sequences for further in-vivo experiments.

### 2.6. Intra-Pulmonary Delivery of sh-lincRNAp21 in Pristane-Induced Model of DAH

**Figure 6:**
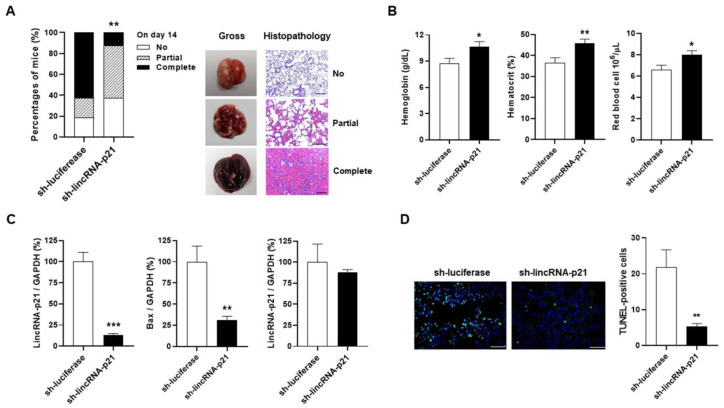
Intra-pulmonary delivery of sh-lincRNAp21 in pristane-induced model of DAH. (**A**) Pulmonary hemorrhagic frequencies of sh-luciferase- and sh-lincRNAp21-treated mice on day 14 (left panel). Representative gross and histopathology findings in the lungs with no, partial and complete hemorrhage (right panel). (**B**) Hb, Hct and RBC of sh-luciferase- and sh-lincRNAp21-treated mice on day 14. (**C**) LincRNA-p21 expression levels in the lungs (left panel) and spleen (right panel) from sh-luciferase- and sh-lincRNAp21-treated mice on day 14. Bax expression levels in the lungs (middle panel) from sh-luciferase- and sh-lincRNAp21-treated mice on day 14. (**D**) Representative TUNNEL staining in lung tissues from sh-luciferase- and sh-lincRNAp21-treated mice (left panel, ×400). Bars shown on photomicrographs corresponding to 20 μm. Numbers of TUNEL-positive cells (right panel), as determined by averaging the number from 3 fields (×400) of the highest density of positively stained cells in each section. Relative abundance of a measured gene expression was normalized by GAPDH gene from each sample. The average levels of lung and spleen tissues from sh-luciferase-treated mice on day 14 were determined as 100%. Values are the mean ± SEM with 16 mice per group in (**A**,**B**), and 8 mice per group in (**C**,**D**). All of the in vivo results in Figure 6 were representative of two independent experiments with similar findings. * *p* < 0.05, ** *p* < 0.01, *** *p* < 0.001.

Only DAH occurred without the proteinuria manifestation on day 14 after pristane injection, indicating the lungs as a main target organ in this mouse model. After IT administration of sh-lincRNAp21 or sh-luciferase, mice were injected with pristane and sacrificed on day 14 for evaluating the therapeutic effects by gross examinations and histopathological analyses. sh-lincRNAp21-injected mice had significantly lower complete hemorrhage frequencies than sh-luciferase-treated controls (Figure 6A, 12.5 versus 62.5%, *p* = 0.009). Furthermore, mice receiving sh-lincRNAp21 therapy had improved anemia status with significantly higher Hb levels, hematocrit (Hct) percentages and RBC numbers than those under the sh-luciferase treatment (Figure 6B, Hb, 8.8 ± 0.6 versus 10.6 ± 0.6 g/dL, *p* = 0.030; Hct, 36.5 ± 2.5 versus 45.9 ± 1.8%, *p* = 0.005; RBC, 6.6 ± 0.4 versus 8.0 ± 0.4 10^6^/μL, *p* = 0.017). We further examined lincRNA-p21 expression in the lungs and spleen upon sacrifice on day 14. There were significantly lower pulmonary levels in sh-lincRNAp21-treated mice than in sh-luciferase-treated controls (Figure 6C, 12.9 ± 2.1 versus 100.0 ± 10.9%, *p* < 0.001), but no differences in splenic levels between two treatment groups.

LincRNA-p21 is transcriptionally activated by p53, and this molecule can provide a feedback to enhance the p53 transcriptional activity and increase the expression of its downstream genes [24,25]. We examined the Bax expression, and identified significantly lower pulmonary levels in mice receiving sh-lincRNAp21 treatment than under sh-luciferase administration (Figure 6C, 30.9 ± 4.7 versus 100.0 ± 18.6%, *p* = 0.007). Furthermore, there were significantly lower TUNEL-positive cell numbers in lung tissues from sh-lincRNAp21-treated mice than from sh-luciferase-treated controls (Figure 6D, 5.3 ± 0.9 versus 21.9 ± 4.8, *p* = 0.004).

Altogether, these in vivo evidences verified the therapeutic potential of targeting intra-pulmonary p53-dependent lncRNA in SLE-associated DAH.

## 3. Discussion

Current medications are associated with unsatisfying clinical responses in SLE-associated DAH patients, including azathioprine, cyclophosphamide, cyclosporine, intravenous Igs, mycophenolate mofetil and pulse methylprednisolone in addition to the routine prescription of high-dose corticosteroids [1,3,16]. Although therapeutic plasmapheresis has been widely applied in refractory cases, this procedure has inherent risks of adverse events and no beneficent impact on the survival [1,30,31]. Since the activation of B cells participates in the clinical activity of SLE-associated DAH [1,3], rituximab (B-cell depleting monoclonal antibody) has been used as an alternative therapeutic agent to cyclophosphamide in such patients with beneficial effects [8,10]. Nevertheless, a multicentric series included RTX as a therapeutic regimen in selected patients with a mortality outcome in all [7]. Based on these inconsistent observations, large-scale randomized trials are needed to elucidate the therapeutic efficacy of RTX in SLE-associated DAH. Interestingly, local pulmonary infusion of activated recombinant factor VII via the administration of nebulizer or bronchoscopy has been demonstrated to achieve hemostasis and improve DAH in SLE patients with active disease [1,3,32,33,34]. In this study, intra-pulmonary delivery of shRNA targeting the p53-dependent lncRNA expression could decrease hemorrhage and improve anemia through reducing cell apoptosis in the lungs of a mouse model, implicating the therapeutic potential in clinical patients. Nevertheless, only limited case numbers were enrolled into the present investigation. More numbers of patients are needed in further studies for elucidating the complex mechanisms to help develop novel therapeutics in controlling such a lethal presentation.

In rheumatoid arthritis (RA), an autoimmune disease targeting the joints, p53 is overexpressed in the synovial intimal lining, a primary DNA damage site, and is constitutively expressed by fibroblast-like synoviocytes [35]. Decreased lincRNA-p21 levels has been reported to cause pro-inflammatory cytokines-induced activity by reducing sequestration of NF-κB p65 mRNA in RA T cells [36]. In fibroblast-like synoviocytes from RA patients, increased levels of H19, rather than HOTAIR, can down-regulate the p53 expression to generate apoptosis-resistant status, leading to bone and cartilage erosions with articular destruction [26,37]. Notably, reduced p53 levels have been demonstrated in whole blood cells from RA patients [37], whereas increased p53 expression in circulating lymphocytes and renal parenchymal cells with activated apoptosis has been shown to be involved in the SLE pathogenesis with a clinical activity correlation [20,21,22,23]. In this study, SLE-associated DAH patients had higher levels of lincRNA-p21, instead of H19 and HOTAIR, in circulating MNCs, and its expression was increased in lung parenchymal cells by ISH analysis. These experimental results indicate that, in effector cells from different autoimmune disorders with specific target organs, distinctive expression of p53-dependent lncRNA such as lincRNA-p21 in SLE pulmonary cells and H19 in RA synoviocytes can modulate the apoptotic process, resulting in accelerated and receded cell apoptosis, respectively, to perpetuate the disease activity. Interestingly, in the SLE and RA disease states, elevated levels of pro-inflammatory cytokines like tumor-necrosis factor-α and IL-6 have been shown to affect the lncRNA expression in different target organs [37,38,39].

Importantly, lncRNA with microRNA (miRNA) response elements can communicate with others via the miRNA messenger, and serve as competing endogenous RNA with the function to sponge or sequestrate miRNA for degradation [40]. Both mRNAs and lncRNA can be recognized by the same miRNA, and indirectly regulate each other by competing for such molecules. Accumulating evidences have shown that interactions between lncRNA and miRNA can control different cellular processes to affect the clinical states of autoimmune disease [41]. Furthermore, recent investigations have identified a pathogenic role of miRNA in the pristane-induced DAH model of lupus mice [42]. There were up-regulated miR-155 and down-regulated miR-125a expression during the development of DAH with corresponding target molecules peroxisome proliferator-activated receptor α and IL-16, respectively [43,44]. Moreover, intravenous injection of miRNA-155 antagomirs and intraperitoneally infusing miR-125a mimics could reduce the complete hemorrhage frequencies and totally abolish the DAH presentation, respectively. Interestingly, two up-to-date reports examined the roles of lncRNA NEAT1 and TUG1, in the pristane-induced LN model [45,46]. In these mice, NEAT1 deficiency attenuated proteinuria amounts through inhibiting the type I interferon-induced activation of B cells, while NF-κB inhibitor therapy improved serum creatinine levels by reducing the apoptosis of renal cells through enhancing TUG1 expression. In this study, we firstly identified a pathogenic role of lncRNA in SLE-associated DAH patients and their corresponding mouse model. Indeed, these experimental results have provided potential miRNA and lncRNA molecule targets for treating the DAH manifestation in SLE patients.

Since there are difficulties in carrying out patient studies in SLE, a heterogenous clinical disorder with life-threatening presentations, various mouse models have been developed for analyzing disease mechanisms and identifying therapeutic targets [47]. Renal involvement with glomerulonephritis has been shown in different models including F1 hybrid from New Zealand Black and White strains, lpr mutation in Fas gene on MRL background and pristane-injected BALB/C mice driven by a type 1 interferon-mediated response. Among various spontaneous, induced or genetically engineered SLE models, DAH is a unique presentation and occurs only in C57BL/6 and C57BL/10 strains receiving intraperitoneal injection of pristane [42,48]. Comparable with SLE-associated DAH patients, the pristane-induced mouse model has the presence of pulmonary capillaritis, interstitial neutrophilic infiltration and hemosiderin-laden macrophages in their lungs [3,42]. Despite a lack of serial kinetic survey and concurrent IF assay, evident electron-dense materials observed by electron microscopy were absent in alveolar basement membrane and vascular endothelial cells in pristane-injected C57BL/10 mice on day 14 [49]. Owing to a rapid disease course in DAH mice without earlier appearance of circulating autoantibodies (e.g., anti-RNP in our results), it has been suggested that its mechanisms involve opsonization of pristane-induced apoptotic cells by IgM and C3, followed by the complement receptor-mediated inflammation responses [16,50]. Nevertheless, the requirement of Igs and complements for the development of DAH after pristane injection indicates an essential role of ICs in this mouse model, highlighting the similarities in the SLE-associated DAH pathogenesis between patients and mice [2,16,42,50]. Furthermore, the occurrence of DAH was not reduced in pristane-injected C57BL/6 lpr mice [42], and immune effector cells from such mice were still sensitive to in vitro hydrophylic pristane-induced apoptosis [15], specifying that pristane-induced cell apoptosis is independent of Fas-mediated extrinsic pathway. In addition, hydrophylic pristane-induced T cell apoptosis with the release of cytochrome c could be blocked by a caspase 9 inhibitor, indicating the involvement of intrinsic mitochondrial pathway [15,51]. Taken together, these available in-vitro and in-vivo results, as well as our experimental findings, have indicated the participation of mitochondrial p53-related apoptosis pathway in the pathophysiology of SLE-associated DAH.

In this study, mice receiving LV-mediated shRNA targeting intra-pulmonary lincRNA-p21 expression had lower complete hemorrhage frequencies and improved anemia status (Figure 6A,B), indicating a success of the IT administration in treating the SLE-associated DAH presentation. In CD4+ splenocytes obtained after pristane injection, there were higher lincRNA-p21 levels with increased expression of apoptosis-related molecules and apoptotic cell ratios (Figure 4A,D), bringing about a possibility that systemic administration of sh-lincRNA-p21 LV vectors through intravenous injection to suppress the development of apoptotic cells outside the lungs could improve the therapeutic efficacy. Nevertheless, by comparing sh-lincRNA-p21- and sh-luciferase-treated groups, despite lower linRNA-p21 levels in the lungs from mice receiving sh-lincRNA-p21 therapy, there were no differences in the spleen (Figure 6C), suggesting no evident extra-pulmonary targeting effects related to the local IT delivery. In our earlier experiments in rodent arthritis models, intra-articular injection of LV vectors targeting or overexpressing lncRNA or miRNA molecules, also revealed no extra-articular global impacts by this regional administration route [37,52,53]. Since safety is a major concern in using LV vectors for clinical therapy, the local delivery can minimize the undesired adverse effects to non-target organs through the systemic administration with intravenous injection [54].

## 4. Materials and Methods

### 4.1. SLE Patients and Age/Sex-Matched HC Subjects

Fifteen patients fulfilling the American College of Rheumatology revised Criteria for SLE [55], 12 females and 3 males aged from 25 to 54 years (35.3 ± 8.7), and 15 age/sex matched HC subjects were enrolled into this study. Medical records were reviewed for their demographic features, clinical profiles including disease period, involved organ number and SLE disease activity 2000 (SLEDAI-2K) and therapeutic modalities (Table 2). DAH was defined as new bilateral pulmonary infiltrates on images, an abrupt drop of Hb levels at least 1.5 g/dL without bleeding elsewhere, and at least one of the following presentations: hemoptysis, hypoxemia, bloody appearance in bronchoalveolar lavage fluid and histopathological evidence of alveolar hemorrhage [10].

In this study, 5 SLE patients had the DAH complication (DAH group) according to the DAH definition in this study. Five age/sex matched patients had LN with the class IV histopathological proof (LN group). There was neither DAH nor LN in another 5 age/sex matched patients (Nil group). All patients received the corticosteroids treatment. The therapeutic profiles in DAH and LN groups included the use of cyclophosphamide, azathioprine and mycophenolate mofetil. Rituximab infusion were prescribed for both DAH and LN groups. Plasmapheresis therapy was prescribed in one DAH patient. In the Nil group, two patients received the azathioprine treatment. There were no differences between sex/age and disease periods among different patient groups. Venous blood samples were collected from SLE patients and HC subjects. Fresh urine specimens were obtained from LN and DAH patients and healthy controls. Although lung biopsy is rarely necessary [1], lung tissues were obtained from two SLE-associated DAH patients for the differential diagnosis of infection complications and pathogens identification due to no conclusive results from their bronchoalveolar lavage survey. Pulmonary specimens from pneumothorax (PTX), a non-inflammatory disorder without underling lung diseases, were served as the control. This study was approved by the Institutional Review Board of National Cheng Kung University Hospital (NCKU Hospital, approval number A-ER-108-455) with the informed consent from each enrolled patient.

### 4.2. Purification of Human and Mouse Cells

Human MNCs were isolated from venous blood samples by Ficoll-Paque PLUS (GE Healthcare, Chicago, IL, USA), and urine cell pellets were obtained from fresh urine specimens by centrifugation and washing procedures. Mouse spleens were homogenized by using syringe plunger and mesh strainer to collect splenocytes. Human MNCs were incubated with PE/Cy5 anti-human CD4 (BD Pharmingen, San Diego, CA, USA) and FITC anti-human CD14 (BD Pharmingen) antibodies, and mouse splenocytes were incubated with PE/Cy5 anti-mouse CD4 (BD Pharmingen) and FITC anti-mouse CD19 (BD Pharmingen) antibodies. These cells were sorted by Moflo XDP Cell Sorter (Beckman Coulter, Mountain View, CA, USA) to obtain human CD4+ and CD14+ cells and mouse CD4+ and CD19+ cells with the purity up to 95%.

### 4.3. Pristane-Induced Model of DAH

Eight-week-old female C57BL/6 Jackson National Applied Research Laboratories mice were purchased from the National Laboratory Animal Center (Taipei, Taiwan), and housed under specific pathogen-free conditions in the Laboratory Animal Center of NCKU. Animal experiments were approved by the NCKU Institutional Animal Care and Use Committee, and performed according to its guidelines. Mice received intraperitoneal injection of 0.5 mL pristane (Sigma-Aldrich, St. Louis, MO, USA) to induce DAH, while their controls were injected with 0.5 mL of PBS [48,50]. They were sacrificed on day 14 to obtain the lungs and spleen. In addition, their urine specimens were acquired on day 14, and blood samples were collected periodically.

### 4.4. Quantitative Real Time Polymerase Chain Reaction (qRT-PCR) Examination

The total RNAs from human or mouse cells and mouse tissues were extracted by TRIzol reagent (Invitrogen Carlsbad, CA, USA), and complementary DNAs were obtained by using reverse transcriptase (Applied Biosystems, Foster City, CA, USA). qRT-PCR was performed to quantify the target RNAs levels by using the SYBR qPCR Mix Kit (TOOLS) [53]. The condition of PCR was: 95 °C for 5 min, 95 °C for 15 s, primer melting temperature (Tm) for 1 min with 40 cycles, and elongation at 72 °C for 20 s. Primer sequences were as follows.

Human lincRNA-p21 (Tm 59 °C), forward 5′-GTGCAGAGCGTTTTGTTTGTCCAT-3′/reverse 5′-C CACAGCCTCTGGGAAGAAAATG-3′.

Human HOTAIR (Tm 60 °C), forward 5′-GTGTAGACCCAGCCCAATTTA-3′/reverse 5′-GGCTGG ACCTTTGCTTCTAT-3′.

Human H19 (Tm 57 °C), forward 5′-GAAATGCTACCCAGCTCAAGC-3′/reverse 5′-CTGCTGTTCC GATGGTGTCTTTGA-3′.

Human GADPH (Tm 54 °C), forward 5′-ACTTCAACAGCACACCCACT-3′/reverse 5′-GCCAAATT CGTTGTCATACCAG-3′.

Mouse lincRNA-p21 (Tm 57 °C), forward 5′- CCGACAGGAGTCTCATGCTCAG -3′/revers 5′-CTGACCCAGAC CAGTCTGGGC-3′.

Mouse p53 (Tm 56 °C), forward 5′-TGAACCGCCGACCTATCCTTA-3′/reverse 5′-GGCACAAACA CGAACCTCAAA-3′.

Mouse Bax (Tm 59 °C), forward 5′-AGGATGCGTCCACCAAGAAGCT-3′/reverse 5′-TCCGTGTCC ACGTCAGCAATCA-3′.

Mouse GADPH (Tm 56 °C), forward 5′-GTTGTCTCCTGCGACTTCAACA-3′/reverse 5′-TTGCTGT AGCCGTATTCATTGTC-3′.

Relative abundance of a measured gene expression was normalized by GAPDH gene from each sample. The average levels of human MNCs or urine cells from HC subjects, and mouse splenocytes or lung tissues on day 0, purified cell subpopulations of PBS-injected mice on day 14, lung and spleen tissues from sh-luciferase-treated mice on day 14, and expression levels of alveolar epithelial cells without stimulation or control transfectants were determined as 100%.

### 4.5. Hydrophilic Pristane Preparation

To study the biological responses of pristane on in vitro cultured cells was hindered by the extreme hydrophobicity, being solved by using an inclusion complex of pristane and β-CyD to circumvent the poor immiscibility of oils with aqueous culture media [56]. For the formation of such complexes, 4 mM solution of β-CyD (Sigma-Aldrich) was mixed with pristane, stirred for 4 days at room temperature, and washed with PBS. Finally, the precipitate was re-suspended in PBS, and the concentrations were adjusted by measuring the optical densities at 254 nm by UV spectrometry [56,57].

### 4.6. LV-Based shRNA Targeting LincRNA-p21 Construction

Four shRNA sequences targeting mouse lincRNA-p21 were designed as follows.

#1, sense 5′-GCTTAAGTTTGTTTATTTA-3′, antisense 5′-TAAATAAACAAACTTAAGC-3′.

#2, sense 5′-GGAAGAACGAGCAATTATA-3′, antisense 5′-TATAATTGCTCGTTCTTCC-3′.

#3, sense 5′-GAACTCTGTACTTTAATTA-3′, antisense 5′-TAATTAAAGTACAGAGTTC-3′.

#4, sense 5′-TGAAAAGAGCCGTGAGCTATC-3′, antisense 5′-GATAGCTCACGG CTCTTTTCA-3′.

For cloning shRNA sequences targeting lincRNA-p21, a 1.9 kb stuffer was removed by *Age*I and *Eco*RI (New England Biolab, Beverly, MA, USA) from pLKO.1-puro (National RNAi Core Facility, Academia Sinica, Taipei, Taiwan), a LV vector containing a woodchuck hepatitis virus posttranscriptional regulatory element to enhance the gene expression levels [58,59]. Luciferase shRNA-expressing pLKO.1-sh-luciferase LV plasmids from the National RNAi Core Facility (Academia Sinica) were used as a scramble control vector in this study. To obtain recombinant LV vectors, the created pLKO.1-sh-lincRNA-p21 and obtained pLKO.1-sh-luciferase expressing vectors were transiently transfected into sub-confluent HEK 293T cells (American Type Culture Collection, Manassas, VA, USA), along with the packaging psPAX2 and envelope pMD2.G plasmids by using calcium phosphate precipitation method to acquire LV-sh-lincRNAp21 and LV-sh-luciferase, respectively [53,60]. After transfection for 48 to 72 h, cell supernatants were harvested and stored at −80 °C until use. LV titers expressed as viral particles (VPs) were determined by analyzing the virus-associated p24 core protein (QuickTiter Lentivirus titer kit, Cell Biolabs, San Diego, CA, USA). On the basis of the titers of transduction unit (TU) and VP for recombinant LV, 2000 VPs equals to 1 TU [52].

### 4.7. Stable Transfectant Preparation for Screening LincRNA-p21 Knockdown Efficacy

Alveolar epithelial cells (MLE-12, American Type Culture Collection) with 5 × 10^5^ cells/mL in 6-well plate, were infected with LVsh-lincRNAp21 or LV-sh-luciferase for 48 h in the presence of polybrene (8 μg/mL, Sigma-Aldrich), and further incubated with puromycin (2 μg/mL, Sigma-Aldrich) [60]. The selection process was around 2 weeks to select successfully transduced stable cells. The transfectants were seeded with 1 × 10^6^ cells/mL in 6-well plate without or in the presence of various concentrations of Dox (TTY Biopharm, Taiwan) under 37 °C, 5% CO_2_ incubation for 24 h, and further examined for lincRNA-p21 expression by qRT-PCR analysis and for apoptotic cell ratios by flow cytometric assay.

### 4.8. In Vitro Hydrophylic Pristane Stimulation

MLE-12 cells (American Type Culture Collection) were seeded with 1 × 10^6^ cells/mL in 6-well plate in the presence of different concentrations of β-CyD-pristane complex or 1 µM Dox (TTY Biopharm) for 24 h under 37 °C, 5% CO_2_ incubation. After the culture for 24 h, these cells were subjected to qRT-PCR analysis for lincRNA-p21 levels and flow cytometric assay for apoptotic cell ratios.

### 4.9. LV-sh-lincRNAp21 IT Administration and Therapeutic Evaluation

Mice were anesthetized and then received 5 × 10^10^ VPs of LV-sh-lincRNAp21 or LV-sh-luciferase by IT delivery of fluid bolus into the posterior oropharynx above the entrance of trachea [61]. Aspiration of LV solution was confirmed in each recipient mouse by visualization of two operators. These mice received intraperitoneal injection of 0.5 mL pristane on the next day. DAH was evaluated upon sacrifice on day 14 after pristane injection. Hemorrhage frequencies in the lungs were classified according to histopathological findings and gross examinations into no, partial or complete hemorrhage [48,62].

### 4.10. Hemogram, RNP Antibody and Proteinuria Determination in Mice

Upon sacrifice on day 14, anticoagulated blood samples were measured for Hb, Hct and RBC by an automatic blood cell analyzer (Scil Vet Focus 5, Scil Animal Care, Germany). After the injection of pristane or PBS, serum samples from mice were periodically examined for the presence of anti-RNP levels with a mouse anti-RNP enzyme-linked immunosorbent assay kit (Alpha Diagnosis, San Antonio, TX, USA). Urine samples were collected on day 14, and proteinuria was analyzed by urine testing strips (Arkray, Edina, MN, USA).

### 4.11. Cell Apoptosis Measurement

MLE-12 cells were stained with PE-Annexin V (BD Pharmingen) and 7-amino-actinomycin D (BD Pharmingen). Splenocytes from PBS- and pristane-injected mice were stained for FITC-conjugated CD4 (BD Pharmingen), PE-Annexin V and 7-amino-actinomycin D. These stained cells were analyzed by flow cytometry. Annexin V-positive and 7-amino-actinomycin D-negative cells were defined as apoptotic cells. The average apoptotic percentages of MLE-12 cells without stimulation, or CD4+ splenocytes from PBS-injected mice on day 14, were determined as 1.0 (apoptotic cell ratios).

### 4.12. Immunoblotting Assay

Cell lysates were separated by electrophoresis on 10 to 15% SDS-PAGE, transferred on PVDF membranes (Merck Millipore, Burlington, MA, USA), blocked in 5% of non-fat dry milk, and incubated with primary antibodies including anti-caspase 3 (Cell signaling, Danvers, MA, USA), anti-procaspase 3 (Cell signaling), anti-p21 (Santa Cruz, Santa Cruz, CA, USA), or anti-β-actin antibodies (Sigma-Aldrich) at 4 °C for 18 h [53,60]. After washing, the membranes were further incubated with secondary antibodies (Jackson Immunoresearch, West Grove, PA, USA) at room temperature for 2 h. Signal expression of protein-antibody complexes was detected by ECL system (Amersham Pharmacia Biotech, UK) and visualized with Biospectrum imaging system (UVP, Upland, CA, USA). The relative protein expression levels were measured by Image J (National Institutes of Health, Bethesda, MD, USA).

### 4.13. H&E, TUNEL and IF Histopathological Staining

Removed lung tissues were fixed in 10% buffered formalin overnight, embedded in paraffin, cut into 5 µm sections, and stained with H&E.

Paraffin-embedded sections were de-paraffinized in xylene, dehydrated in ethanol and rehydrated in distilled water. For TUNEL assay to detect apoptotic cells, antigens in the lung tissues were reactivated by proteinase K, re-fixed by 4% formaldehyde, incubated with equilibrate buffer, and finally labelled by the TUNEL detection cocktail (Promega, Madison, WI, USA). Number of TUNEL-positive cells, as determined by averaging the number from 3 fields (×400 magnification) of the highest density of positively stained cells in each section observed by fluorescence microscopy [63]. Cell nuclei were counterstained with 4,6-diamidino-2-phenylindole (DAPI) (Sigma-Aldrich).

For detection of TUNEL and CD4 double-positive cells, after labelled with TUNEL reagents, mouse lung sections were stained with anti-mouse CD4 antibodies (BD Biosciences, San Jose, CA, USA), followed by Texas Red–conjugated secondary antibodies (BD Biosciences). The IF staining was observed under the confocal microscopy [60].

For detection of p53 nuclear expression, de-paraffinized lung sections were stained with antibodies against p53 (Santa Cruz), followed by Alexa Fluor 488-conjugated secondary antibodies (Thermo Fisher Scientific, Waltham, MA, USA). Number of p53-positive cells, as determined by averaging the number from 3 fields (×400 magnification) of the highest density of positively stained cells in each section observed by fluorescence microscopy [63]. Cell nuclei were counterstained with DAPI (Sigma-Aldrich).

### 4.14. ISH Assessment

Pretreated de-paraffinized sections were subjected to acetylation solution, fixed with paraformaldehyde, and hybridized in hybridization buffer (Qiagen, Germantown, MD, USA) containing digoxigenin (DIG)-labeled lincRNA-p21 antisense probes, 5′-TGACTTATGATGGTTCAGCTT-3′ or 5′-AGGTCTGAATGTAAGTTGTCT-3′ (Exiqon, Denmark). The scramble probe was used as a negative control (miRCURY LNA detection probes, Exiqon). These sections were then incubated with alkaline phosphatase-conjugated anti-DIG antibody, and finally stained with 5-bromo-4-chloro-3-indolyl-phosphate/nitroblue tetrazolium chromogen solution [53]. Cell nuclei were counterstained with Nuclear Fast Red solution (Sigma-Aldrich).

### 4.15. Statistical Analyses

Data are expressed as the mean ± standard error of the mean. The expression levels of lncRNA between SLE patients and HC subjects or different patient groups were analyzed by Mann-Whitney U test. Correlation analysis was done by Spearman correlation coefficient test with linear regression analysis. The differences in other in vitro analyses were determined by Student’s *t* test. Different complete hemorrhage frequencies between sh-lincRNAp21- and sh-luciferase-treated mouse groups were compared by the Fisher’s exact test. *p* values less than 0.05 were considered significant in this study with symbols presenting as * for *p* < 0.05, ** for *p* < 0.01 and *** for *p* < 0.001.

## 5. Conclusions

In SLE patients, the DAH manifestation has a significant mortality, requiring a thorough understanding of its complex pathogenesis to develop novel therapeutics for disease control. In this study, there was increased expression of p53, lincRNA-p21 and cell apoptosis in the lungs of SLE-associated DAH patients and mice. Intra-pulmonary delivery of shRNA targeting lincRNA-p21 reduced hemorrhage frequencies with improved anemia status through decreasing Bax expression and cell apoptosis in the mouse model. Our findings demonstrate for the first time the therapeutic potential of targeting p53-dependent lncRNA expression in SLE-associated DAH.

## Figures and Tables

**Table 1:** Larger-scale series of SLE-associated DAH patients in the recent decade *.

No.	Source	Case No.	Incidence	^#^ Age/Sex	CYC	MP	TP	IVIG	RTX	MR	Ref
1	2011 ROK	21	1.4%	30/F 91%	38%	NR	67%	38%	Nil	62%	4
2	2011 Mexico	22	9.0%	25/F 76%	59%	NR	0%	5%	9%	68%	5
3	2012 Brazil	28	1.6%	23/F 82%	57%	100%	18%	25%	Nil	39%	6
4	2014 Mexico	50	NR	23/F 98%	49%	NR	0%	8%	6%	48%	7
5	2015 United States	22	2.2%	37/NR	41%	45%	18%	14%	14%	0% ^@^	8
6	2017 ROK	24	2.9%	30/F 100%	17%	NR	33%	50%	Nil	29%	9
7	2018 Taiwan	17	2.0%	38/F 88%	35%	71%	43%	Nil	24%	35%	10
8	2018 Brazil	19	2.2%	13/F 74%	47%	95%	0%	Nil	Nil	47%	11
9	2020 Colombia	17	NR	28/F 65%	77%	100%	100%	Nil	6%	29%	12
10	2020 China	94	2.0%	29/F 87%	76%	79%	38%	71%	Nil	38%	13

* Case series with patient no. more than 10, ^#^ Average age of reported patients. ^@^ No acute death but a 27% MR in the later period higher than control patients. CYC: cyclophosphamide, F: female, IVIG: intravenous immunoglobulin, MP: methylprednisolone pulse, MR: mortality rate, No.: number, NR: not reported, Ref: reference, ROK: Republic of Korea, RTX: rituximab, TP: therapeutic plasmapheresis.

**Table 2:** Demographic features, clinical characters and therapeutic profiles of enrolled SLE patients.

Patient Group	Nil	LN	DAH	*p* Values
**Demographic** **f** **eature**				
Patients number	5	5	5	
Age (year)	35.4 ± 7.3	34.6 ± 9.3	36.0 ± 11.1	NS
Female	4 (80%)	4 (80%)	4 (80%)	NS
**Clinical profile**				
Disease period (years)	5.4 ± 3.4	5.2 ± 4.3	4.6 ± 3.7	NS
Activity score (SLEDAI-2K)	2.2 ± 1.5	8.6 ± 2.4	19.2 ± 3.7	* *p* < 0.01
Involved organ number	0.6 ± 0.5	2.8± 0.4	3.8 ± 0.4	* *p* < 0.01
**Therapeutic modality**				
Corticosteroid	5 (100%)	5 (100%)	5 (100%)	NS
Cyclophosphamide	0 (0%)	4 (80%)	4 (80%)	* *p* < 0.05
Mycophenolate mofetil	0 (0%)	3 (60%)	2 (40%)	NS
Azathioprine	2 (40%)	2 (40%)	3 (60%)	NS
Rituximab	0 (0%)	1 (20%)	2 (40%)	NS
Plasmapheresis	0 (0%)	0 (0%)	1 (20%)	NS
Mechanic ventilator	0 (0%)	0 (0%)	5 (100%)	^#^*p* < 0.01
ECMO	0 (0%)	0 (0%)	1 (20%)	NS

DAH: diffuse alveolar hemorrhage, ECMO: extracorporeal membrane oxygenation, LN: lupus nephritis, NS: not significant, SLE: systemic lupus erythematosus. * DAH or LN versus Nil, ^#^ DAH versus LN or Nil.

## Data Availability

The data of this study can be provided to researchers from the corresponding author upon reasonable request.

## Abbreviations

β-CyD	β-cyclodextrin
DAH	Diffuse alveolar hemorrhage
DAPI	4,6-diamidino-2-phenylindole
DIG	Digoxigenin
Dox	Doxorubicin
GN	Glomerulonephritis
Hb	Hemoglobulin
Hct	Hematocrit
IC	Immune complexes
IF	Immunofluorescence
Ig	Immunoglobulin
LncRNA	Long noncoding RNA
LN	Lupus nephritis
LV	Lentivirus
MiRNA	MicroRNA
MNC NCKU	Mononuclear cell National Cheng Kung University
PBS	Phosphate-buffered saline
qRT-PCR	Quantitative real-time polymerase chain reaction
RBC	Red blood cell
RT RA	Reverse transcription Rheumatoid arthritis
SEM ShRNA	Standard error of the mean Short hairpin RNA
SLE	Systemic lupus erythematosus
SLEDAI	SLE disease activity index
Tm	Temperature for primer melting
TUNEL	Terminal deoxynucleotidyl transferase dUTP nick end labeling
